# Bioinformatic Tools for Inferring Functional Information from Plant Microarray Data II: Analysis Beyond Single Gene

**DOI:** 10.1155/2008/893941

**Published:** 2008-07-03

**Authors:** Issa Coulibaly, Grier P. Page

**Affiliations:** Department of Biostatistics, University of Alabama at Birmingham, 1665 University Blvd Ste 327, Birmingham, AL 35294-0022, USA

## Abstract

While it is possible to interpret microarray experiments a single gene at a time, most studies generate long lists of differentially expressed genes whose interpretation requires the integration of prior biological knowledge. This prior knowledge is stored in various public and private databases and covers several aspects of gene function and biological information. In this review, we will describe the tools and places where to find prior accurate biological information and how to process and incorporate them to interpret microarray data analyses. Here, we highlight selected tools and resources for gene class level ontology analysis (Section 2), gene coexpression analysis (Section 3), gene network analysis (Section 4), biological pathway analysis (Section 5), analysis of transcriptional regulation (Section 6), and omics data integration (Section 7). The overall goal of this review is to provide researchers with tools and information to facilitate the interpretation of microarray data.

## 1. INTRODUCTION

Microarray analysis is exploratory and very high dimensional, and the primary purpose is to generate a list of differentially regulated genes that can provide insight into the biological phenomena under investigation. However, analysis should not stop with a list, it should be the starting point for secondary analyses that aim at deciphering the molecular mechanisms underlying the biological phenotypes analyzed. Combining microarray data with prior biological knowledge is a fundamental key to the interpretation of the list of genes. This prior knowledge is stored in various public and private databases and covers several aspects of genes functions and biological information such as regulatory sequence analysis, gene ontology, and pathway information. In this review, we will describe the tools and places where to find prior accurate biological information and how to incorporate them into the analysis of microarray data. The plant genome outreach portal (http://www.plantgdb.org/PGROP/pgrop.php?app=pgrop) list many of these resources and other tools and resources such as EST resources and BLAST that are not covered in this review. We also address some theoretical aspects and methodological issues of the algorithms implemented in the tools that have been recently developed for bioinformatic and what needs to be considered when selecting a tool for use.

## 2. CLASS LEVEL FUNCTIONAL ANNOTATION TOOLS

The goal of these class level functional annotation tools is to relate the expression data to other attributes such as cellular localization, biological process, and molecular function for groups of related genes. The most common way to functionally analyze a gene list is to gather information from the literature or from databases covering the whole genome. The recent developments in technologies and instrumentation enabled a rapid accumulation of large amount of in silico data in the area of genomics, transcriptomics, and proteomics as well. The gene ontology (GO) consortium was created to develop consistent descriptions of gene products in different databases [1]. The GO provides researchers with a powerful way to query and analyze this information in a way that is independent of species [2]. GO allows for the annotation of genes at different levels of abstraction due to the directed acyclic graph (DAG) structure of the GO. In this particular hierarchical structure, each term can have one or more child terms as well as one or more parent terms. For instance, the same gene list is annotated with a more general GO term such as “cell communication” at a higher level of abstraction, whereas the lowest level provides a more specific ontology term such as “intracellular signaling cascade.”

In recent years, various tools have been developed to assess the statistical significance of association of a list of genes with GO annotations terms, and new ones are being regularly released [3]. There has been extensive discussion of the most appropriate methods for the class level analysis of microarray data [4–6]. The methods and tools are based on different methodological assumptions. There are two key points to consider: (1) whether the method uses *gene sampling* or *subject sampling* and (2) whether the method uses *competitive* or *self-contained* procedures. The subject sampling methods are preferred and the competitive versus self-contained debate continues. Gene sampling methods base their calculation of the *p*-value for the geneset on a distribution in which the gene is the unit of sampling, while the subject sampling methods take the subject as the sampling unit. The latter is more valid for the unit of randomization is the subjects not the genes [7–9].

*Competitive* tests, which encompass most of the existing tools, test whether a gene class, defined by a specific GO term or pathway or similar, is overrepresented in the list of genes differentially expressed compared to a reference set of genes. A *self-contained* test compares the gene set toa fixed standard that does not depend on the measurements of genes outside the gene set. Goeman et al. [10, 11], Mansmann and Meister [7], and Tomfohr et al. [9] applied the self-contained methods.

Another important aspect of ontological analysis regardless of the tool or statistical method is the choice of the reference gene list against which the list of differentially regulated genes is compared. Inappropriate choice of reference genes may lead to false functional characterization of the differentiated gene list. Khatri and Drǎghici [3] pointed out that only the genes represented on the array, although quite incomplete, should be used as reference list instead of the whole genome as it is a common practice. In addition correct, up to date, and complete annotation of genes with GO terms is critical. The competitive and gene sample-based procedures tend to have better and more complete databases. GO allows for the annotation of genes at different levels of abstraction due to the directed acyclic graph (DAG) structure of the GO. In this particular hierarchical structure, each term can have one or more child terms as well as one or more parent terms. For instance, the same gene list is annotated with a more general GO term such as “cell communication” at a higher level of abstraction, whereas the lowest level provides a more specific ontology term such as “intracellular signaling cascade.” It is important to integrate the hierarchical structure of the GO in the analysis since various levels of abstraction usually give different *p*-values. The large number (hundreds or thousands) of tests performed during ontological analysis may lead to spurious associations just by chance, thus correction for multiple testing is a necessary step to take. We present here a nonexhaustive list of tools available that can be used to perform functional annotation of gene list and attempt to compare their functionalities (Table 1). All tools accept input data from *Arabidopsis thaliana*, the most used model organism in plant studies, as well as many animal organism models.

**Onto-Express (OE): http://vortex.cs.wayne.edu/projects.htm#Onto-Express**

Onto-Express is a software application used to translate a list of differentially regulated genes into a functional profile [12, 13]. Onto-Express constructs a profile for each of the GO categories: cellular component, biological process, molecular function, and chromosome location as well. Onto-Express implements hypergeometric, binomial, *𝒳* ^2^ and Fisher's exact tests. The results are displayed in a graphical form that allows the user to collapse or expand GO node and visualize the *p*-values associated with each level of GO abstraction. Onto-Express performs Bonferroni, Holm, Sidak, and FDR corrections to adjust for multiple testing. Users have an option of either providing their own reference gene list or selecting a microarray platform as reference gene list. An extensive list of up to date annotations is provided for many arrays.

**FuncAssociate: http://llama.med.harvard.edu/cgi/func/funcassociate**

FuncAssociate is a web-based tool that characterizes large sets of genes with GO terms using the Fisher's exact test [14]. Among all annotation tools FuncAssociate stands out in that it implements a Monte Carlo simulation to correct for multiple testing. In addition the tools can conduct analysis on ranked list of query genes. Although FuncAssociate supports 10 organisms, it does not provide visualization or level information for the GO annotation.

**SAFE (Significance Analysis of Function and Expression)**

SAFE is a Bioconductor/R algorithm that first computes gene-specific statistics in order to test for association between gene expression and the phenotype of interest [15]. Gene-specific statistics are used to estimate global statistics that detects shifts in the local statistics within a gene category. The significance of the global statistics is assessed by repeatedly permuting the response values. SAFE implements a rank-based global statistics that enables a better use of marginally significant genes than those based on a *p*-value cutoff.

**Global test**

Global test is a Bioconductor/R package that tests the association of expression pattern of a group of genes with selected phenotypes of interest using self-contained methods [10]. The method is based on a penalized regression model that shrinks regression coefficient between gene expression and phenotype toward a common mean. The algorithm allows the users to test biological hypothesis or to search GO databases for potential pathways. The results of gene lists of various sizes can be compared.

**FatiGO+ (Fast Assignment and Transference of Information):http://babelomics2.bioinfo.cipf.es/fatigoplus/cgi-bin/fatigoplus.cgi**

FatiGO+ tests for significant difference in distribution of GO terms between any two groups of genes (ideally a group of interest and a reference set of genes) using a Fisher's exact test for 2 by 2 contingency table [16]. FatiGO+ implements an inclusive analysis in which at a given level in the GO DAG hierarchy, genes annotated with child GO terms take the annotation from the parent. This increases the power of the test. The software returns adjusted *p*-values using the FDR method [17].

**GOToolBox: http://burgundy.cmmt.ubc.ca/GOToolBox/**

GOToolBox identifies over-or under-represented GO terms in a gene set using either hypergeometric distribution-based tests or binomial test [18]. The user has the option of choosing between the total set of genes in the genome as reference or provides his own list of reference genes. The software implements Bonferroni correction to adjust for multiple testing. Its also allows the user to select a specific level of GO abstraction prior to the analysis.

**CLENCH2 (CLuster ENriCHment): http://www.stanford.edu/~nigam/cgi-bin/dokuwiki/doku.php?id=clench**

Clench is used to calculate cluster enrichment for GO terms [19]. The program accepts two lists of genes: a reference set of genes and the list of changed genes. CLENCH performs hypergeometric, binomial and *𝒳* ^2^ tests to estimate GO terms enrichment. The program allows the user to choose an FDR cutoff in order to account for multiple testing.

**BiNGO (Biological Network Gene Ontology tool): http://www.psb.ugent.be/cbd/papers/BiNGO/**

BiNGO is a Java-based tool to determine which gene ontology (GO) categories are statistically overrepresented in a set of genes or a subgraph of a biological network [20]. BiNGO is implemented as a plugin for Cytoscape, which is an open source bioinformatics software platform for visualizing and integrating molecular interaction networks. The program implements hypergeometric test and binomial test and performs FDR to control multiple testing. BiNGO maps predominant functional themes of the tested genes on the GO hierarchy. It allows a customizable visual representation of the results. One limitation is that the user can only choose between the whole genome or the network under study as reference set of gene for the enrichment test.

**GoSurfer: http://bioinformatics.bioen.uiuc.edu/gosurfer/**

GoSurfer is used to visualize and compare gene sets by mapping them onto gene ontology (GO) information in the form of a hierarchical tree [21]. Users can manipulate the tree output by various means, like setting heuristic thresholds or using statistical tests. Significantly important GO terms resulting from a *𝒳* ^2^ test can be highlighted. The software controls for false discovery rate.

## 3. GENE COEXPRESSION ANALYSIS TOOLS

In most microarray studies, gene expressions are measured on a small number of arrays or samples; however, large collections of arrays are available in microarray database that contain transcript levels data from thousands of genes across a wide variety of experiments and samples. These tools provide scientists with the opportunity to analyze the transcriptome by pooling gene expression information from multiple data sets. This meta-analytic approach allows biologists to test the consistency of gene expression patterns across different studies. Most importantly, the analysis of concerted changes in transcript levels between genes can lead to biological function discovery. It has been demonstrated that genes which protein products cooperate in the same pathway or are in a multimeric protein complex display similar expression patterns across a variety of experimental conditions [22, 23]. Using the guilt-by-association principle, investigators can functionally characterize a previously uncharacterized gene when it displays expression pattern similar to that of known genes. The coexpression relationship between two genes is usually assessed by computing the Pearson's correlation coefficient or other distance measures. Prior to the coexpression analysis, a set of “bait-genes” is selected based on previous biological or literature information. Then the genes which expression is significantly correlated with bait-genes expression are analyzed to identify new potential actors in a given pathway or biological process. However, coexpression between two genes does not necessarily translate into similar function between both genes. Some statistically significant correlations may occur by chance. Some authors suggest that to be sustainable the gene coexpressions observed in one species should be confirmed in other evolutionary close species [24]. Tools have been developed that make use of the large sample size available in these databases to identify more reliable concerted changes in transcripts levels as well as to examine the coordinated change of gene expression levels.

**Cress-express: http://www.cressexpress.org/**

Cress-express estimates the coexpression between a user-provided list of genes and all genes from Affymetrix Ath1 platform using up to 1779 arrays. Cress-express also performs pathway-level coexpression (PLC) [25]. PLC identifies and ranks genes based on their coexpression with a group of genes. Cress-express also delivers results in “bulk” formats suitable for downstream data mining via web services. The tool generates files for easy import into Cytoscape for visualization. The tool has the data processed with a variety of image processing methods: RMA, MAS5, and GCRMA. Investigators can select which of over 100 experiments to include in coexpression analysis.

**ATTED-II (*Arabidopsis thaliana* transfactor and cis-element prediction database): http://www.atted.bio.titech.ac.jp/**

ATTED-II provides coregulated gene relationships in *Arabidopsis thaliana* to estimate gene functions. In addition, it can predict overrepresented cis-elements based upon all possible heptamers. There is also several visualization tools and databases of annotations attached to the coexpression.

**Genevestigator: http://www.genevestigator.ethz.ch/**

Genevestigator is a web-based discovery tool to study the expression and regulation of genes, pathways, and networks [26, 27]. Among other applications, the software allows the user to look at individual gene expression or group of genes coexpression in many different tissues, at multiple developmental stages, or in response to large sets of stimuli, diseases, drug treatments, or mutations. In addition, electronic northern blots and other analyses may be conducted.

**BAR (the botany array resource) expression ANGLER: http://www.bar.utoronto.ca/**

The expression anger allows the user to identify genes with similar expression profile with the user provided gene across multiple samples [28]. The user can specify the Pearson correlation coefficient threshold and the array database to use for the coexpression analysis.

**AthCor@CSB.DB (*A. thaliana* coresponse database): http://csbdb.mpimp-golm.mpg.de/csbdb/dbcor/ath.html**

AthCor is a coexpression tool that allows the use of functional ontology filter to identify genes coexpressed with a gene of interest filtering the search by functional ontologies [29]. The user can select between parametric and nonparametric correlation tests.

**PLEXdb (Plant Expression Database): http://www.plexdb.org/**

PLEXdb serves as a comprehensive public repository for gene expression for plants and plant pathogens [30]. PLEXdb integrates new gene expression datasets with traditional genomics and phenotypic data. The integrated tools of PLEXdb allow plant investigators to perform comparative and functional genomics analyses using large-scale expression data sets.

**ACT (Arabidopsis Coexpression Data mining Tool): http://www.arabidopsis.leeds.ac.uk/act/index.php**

ACT estimates the coexpression of 21 891 *Arabidopsis* genes based on Affymetrix ATH1 platform using a simple correlation test [31]. The web server includes a database that stores precalculated correlation results from over 300 arrays of the NASC/GARNet dataset. A “clique finder” tool allows the user to identify groups of consistently coexpressed genes within a user-defined list of genes. The identification of genes with a known function within a cluster allows inference to be made about the other genes. Users can also visualize the coexpression scatter plots of all genes against a group of genes.

## 4. GENE NETWORK ANALYSIS

Genes and their protein products are related to each other through a complex network of interactions. In higher metazoa, on average each gene is estimated to interact with five other genes [32], and to be involved in ten different biological functions during development [33]. On a molecular level, the function of a gene depends on its cellular context, and the activity of a cell is determined by which genes are being expressed and which are not and how they interact with each other. In such high interconnectedness, analyzing a network as a whole is essential to understanding the complex molecular processes underlying biological systems. The traditional reductionist approach that investigates biological phenomena by analyzing one gene at a time cannot address this complexity. By using systems biology approach and network theories, investigators can analyze the behavior and relationships of all of the elements in a particular biological system to arrive at a more complete description of how the system functions [34]. High-throughput gene expression profiling offers the opportunity to analyze gene interrelationships at the genome scale. Clustering analysis on microarray expression data only extracts lists of coregulated genes out of a large-scale expression data. It does not tell us who is regulating whom and how. However, the task of modeling dynamic systems with large number of variables can be computationally challenging. In gene regulatory networks, genes, mRNA, or proteins correspond to the network nodes and the links among the nodes stand for the regulatory interactions (activations or inhibitions). In this section, we will describe some of the methods and tools used to reconstruct, visualize, and explore gene networks.

### 4.1. Gene network reconstruction algorithms

Two main approaches have been used to develop models for gene regulatory networks [35]. One method is based on Bayesian inference theory which seeks to find the most probable network given the observed expression patterns of the genes to be included in the network. The regulatory interactions among genes and their directions are derived from expression data. Several network structures are proposed and scored on the basis of how well they explain the data as it has been successfully implemented in yeast [36]. The second approach is based on “mutual information” as a measure of correlation between gene expression patterns [37]. A regulatory interaction between two genes is established if the mutual information on their expression patterns is significantly larger than a *p*-threshold value calculated from the mutual information between random permutations of the same patterns. Unlike the Bayesian theory, which tries out whole networks and selects the one that best explains the observed data, the mutual information method constructs a network by selecting or rejecting regulatory interactions between pairs of genes. This method does not provide the direction of regulatory interactions. We present below selected tools that implement either of the aforementioned approaches to reverse-engineer gene regulatory networks.

**BNArray (Bayesian Network Array): http://www.cls.zju.edu.cn/binfo/BNArray/**

BNArray is a tool developed in R for inferring gene regulatory networks from DNA microarray data by using a Bayesian network [38]. It allows the reconstruction of significant submodules within regulatory networks using an extended subnetwork mining algorithm. BNArray can handle microarray data with missing values.

**BANJO (Bayesian Network Inference with Java Objects): http://www.cs.duke.edu/~amink/software/banjo/**

Banjo is a tool developed in Java for inferring gene networks [39]. Banjo implements Bayesian and dynamic Bayesian networks to infer networks from both steady-state and time-series expression data. A “proposer” component of Banjo uses heuristic approaches to search the network space for potential network structures. Each network structure is explored and an overall network's score is computed based on the parameters of the conditional probability density distribution. The network with the best overall score is accepted by a “decider” component of the software. The network retained is processed by Banjo to compute influence scores on the edges indicating the direction of the regulation between genes. The software displays the output network.

**GNA (Genetic Network Analyzer): http://www-helix.inrialpes.fr/article122.html**

GNA is a freely available software used for modeling and simulating genetic regulatory networks from gene expression data and regulatory interaction information [40]. In GNA, the dynamics of a regulatory network is modeled by a class of piecewise-linear differential equations. The biological data are transformed into mathematical formalism. Thus the software uses qualitative constraints in the form of algebraic inequalities instead of numerical values.

**PathwayAssist http://www.ariadnegenomics.com/products/pathway-studio**

PathwayAssist allows the users to create their own pathways by combining the user-submitted microarray expression data with knowledge from biological databases such as BIND, KEGG, DIP [41]. The software provides a graphical user interface and publication quality figures.

### 4.2. Network visualization tools

As a result of the explosion and advances in experimental technologies that allow genome-wide characterization of molecular states and interactions among thousands of genes, researchers are often faced with the need for tools for the visualization, display, and evaluation of large structure data. The main aim of these tools is to provide a summarized yet understandable view of large amount of data while integrating additional information regarding the biological processes and functions. Several network visualization tools have been developed of which we will describe some of the most popular.

**Cytoscape—http://www.cytoscape.org/**

Cytoscape is a general-purpose, open-source software environment for the large scale integration of molecular interaction network data [42]. Dynamic states on molecules and molecular interactions are handled as attributes on nodes and edges, whereas static hierarchical data, such as protein-functional ontologies, are supported by use of annotations. The Cytoscape core handles basic features such as network layout and mapping of data attributes to visual display properties. Many Cytoscape plug-ins extend this core functionality.

**CellDesigner http://www.celldesigner.org/**

CellDesigner is a structured diagram editor for drawing gene-regulatory and biochemical networks based on standardized technologies and with wide transportability to other systems biology markup language (SBML) compliant applications and systems biology workbench (SBW) [43]. Networks are drawn based on the process diagram, with graphical notation system. The user can browse and modify existing SBML models with references to existing databases, simulate and view the dynamics through an intuitive graphical interface. CellDesigner runs on Windows, MacOS X, and Linux.

**VANTED (Visualization and Analysis of Networks with related Experimental Data): http://vanted.ipk-gatersleben.de/**

Vanted is a freely available tool for network visualization that allows users to map their own experimental data on networks drawn in the tool, downloaded from KEGG pathway database, or imported using standard imported formats [44]. The software graphically represents the genes in their underlying metabolic context. Statistical methods implemented in VANTED allow the comparison between treatments or groups of genes, the generation of correlation matrix, or the clustering of genes based on expression pattern.

**Osprey http://biodata.mshri.on.ca/osprey/servlet/Index**

Osprey is a software for visualization and manipulation of complex interaction networks [45]. Osprey allows user defined colors to indicate gene function, experimental systems, and data sources. Genes are colored by their biological process as defined by standardized gene ontology (GO) annotations. As a network complexity increases, Osprey simplifies network layouts through user-implemented node relaxation, which disperses nodes and edges according to anyone of a number of layout options.

**VisANT (Integrative Visual Analysis Tool for Biological Networks and Pathways): http://visant.bu.edu/**

VisANT is a freely available open-source tool for integrating biomolecular interaction data into a cohesive, graphical interface [45–47]. VisANT offers an online interface for a large range of published datasets on biomolecular interactions, as well as databases for organized annotation, including GenBank, KEGG, and SwissProt.

### 4.3. Network exploration tools

One of the main focuses in the postgenomic era is to study the network of molecular interactions in order to reveal the complex roles played by genes, gene products, and the cellular environments in different biological processes. The nodes (genes) of a network can be associated with additional information regarding the gene products, gene positions in the chromosome, or the gene functional annotation. The edges in the network symbolize specific interaction that can be associated with a transcription factor-promoter bond for instance. This information can be automatically retrieved in a number of specialized and publicly accessible databases containing data about the nodes and the interactions. Network exploration tools enable the user to perform analysis on single genes, gene families, patterns of molecular interactions, as well as on the global structure of the network. These tools are able to incorporate both microscale and macroscale analysis using heterogenous data. They can connect to a large number of disparate databases. The user usually has an option to construct interaction networks either by curation or by computation and to associate microarray expression data with known metabolic pathways. Here, we describe some of the most popular network exploration tools.

**MetNet (Metabolic Networking Database): http://www.metnetdb.org/**

MetNet is a publicly available software for analysis of genome-wide mRNA, protein, and metabolite profiling data [48]. The software is designed to enable the biologist to visualize, statistically analyze, and model a metabolic and regulatory network map of Arabidopsis, combined with gene expression profiling data. MetNet provides a framework for the formulation of testable hypotheses regarding the function of specific genes. The tools within MetNet allow the user to map metabolic and regulatory networks; to integrate and visualize data together; to explore and model the metabolic and regulatory flow in the network.

**BiologicalNetworks: http://biologicalnetworks.net/**

BiologicalNetworks is a bioinformatics and systems biology software platform for visualizing molecular interaction networks, sequence and 3D structure information [49]. The tool performs easy retrieval, construction, and visualization of complex biological networks, including genome-scale integrated networks of protein-protein, protein-DNA, and genetic interactions. BiologicalNetworks also allow the analysis and the mapping of expression profiles of genes or proteins onto regulatory, metabolic, and cellular networks.

**PaVESy (Pathway Visualization Editing System): http://pavesy.mpimp-golm.mpg.de/PaVESy.htm**

PaVESy is a data managing system for editing and visualization of biological pathways [50]. The main component of PaVESy is a relational SQL database system that stores biological objects, such as metabolites, proteins, genes, and their interrelationships. The user can annotate the biological objects with specific attributes that are integrated in the database. The specific roles of the objects are derived from these attributes in the context of user-defined interactions. PaVESy can display an individualized view on the database content that facilitates user customization.

**Genevestigator: https://www.genevestigator.ethz.ch**

Genevestigator provides a detailed analysis and navigation through biochemical and/or regulatory pathways. It combines automatically produced or user-created graphical representations of networks (e.g., gene modules or pathways) for the exploratory analysis of a large compendium of gene expression profiles. Effects on gene expression can be projected onto these networks for the following ontologies: anatomy, development, stimulus, and mutation, in form of comparison sets.

## 5. BIOLOGICAL PATHWAY RESOURCES

One of the downstream applications of the reconstruction of a gene regulatory networks or the identification of clusters of functionally related genes is to associate the genes and their interconnections with known metabolic pathways. Biochemists summarized the sequence of enzyme-catalyzed metabolic reactions between biomolecules as a network of interactions that results from the conversion of one organic substance (substrate) to another (product). Depending on the type of interactions analyzed, several types of biochemical networks are identified. These biochemical networks represent the potential mechanistic associations between genes and gene products that are involved in specific biological processes [52]. Because of the curse of dimensionality that sometimes hampers the whole network analysis, investigators often focus on “pathway” rather than “network” when they are investigated a small number of gene interactions. Many specialized databases are available that store and summarize large amount of information on metabolic reactions. Increasingly, identifying and searching the right database is a critical and necessary step in most biological researches. This task can be tedious due to the large number of databases available. For a more comprehensive list of biological pathways resources on the web, the reader is referred to pathguide (http://www.pathguide.org). Following is the list of the most popular pathways resources on the web.

**KEGG (Kyoto Encyclopedia of Genes and Genomes): http://www.genome.jp/kegg**

KEGG aims to link lower-level information (genes, proteins, enzymes, reaction molecules, etc.) with higher-level information (interactions, enzymatic reactions, pathways, etc.). Pathways are included for over 100 species.

**MetaCyc: http://MetaCyc.org/**

MetaCyc is a database of metabolic pathways and enzymes [53]. Its goal is to serve as a metabolic encyclopedia, containing a collection of nonredundant pathways, enzymatic reactions, enzymes, chemical compounds, genes and review-level comments. Enzyme information includes substrate specificity, kinetic properties, activators, inhibitors, cofactor requirements and links to sequence and structure databases. AracCyc (http://www.arabidopsis.org/biocyc/index.jsp) uses MetaCyc as reference database for visualization of *Arabidopsis thaliana* biochemical pathways. Table 2 indicates web links to more online pathways databases.

**BioCarta: http://www.biocarta.com/genes/index.asp**

BioCarta is a web-based resource for exploring biological pathways. BioCarta catalogs pathways, regulation and interaction information for over 120,000 genes covering most model organisms. Data in BioCarta are constantly updated, and new pathways are suggested by the life science research community.

**GeneNeT: http://wwwmgs.bionet.nsc.ru/mgs/gnw/genenet/**

The GeneNet system is designed for formalized description and automated visualization of gene networks [54]. The GeneNet system includes database on gene network components, Java program for the data visualization. GeneNet allows the users to select entities that are involved in the functioning of a particular gene network, to describe the regulatory relations for a particular gene network, and to search for potential transcription factors.

## 6. TRANSCRIPTION REGULATION ANALYSIS TOOLS

Most organisms encode a large number of DNA-binding proteins that act as transcription factors. In *Arabidopsis*, more than 5% of the genes have been estimated to encode transcription factors [55]. Transcription factors bind to short conserved DNA motifs (cis-acting regulatory elements CARE) located at the 5’end of the gene (in a region called promoter) to initiate mRNA transcription. Thus DNA-binding proteins play a key role in all aspects of genetic activity within an organism. They participate in promoting or repressing the transcription of specific genes. Elucidating the mechanisms that underlie the expression of genomes is one of the major challenges in bioinformatics. An interesting hypothesis one might formulate after a successful microarray study is that the genes that are coexpressed may also be coregulated at the transcriptional level. One way to test this hypothesis is to identify overrepresented oligonucleotides sequences as potential binding sites for transcriptions factors in promoter regions of genes clustered in the same group. The statistical test for overrepresentation of regulatory motifs in intergenic regions is the general principle implemented in most algorithms for regulatory motif detection [55]. CAREs can also be predicted through phylogenetic footprinting that is based on sequence similarity between orthologous promoters [56]. Some other approaches have been proposed that integrates comparative, structural, and functional genomics to identify conserved motifs in coregulated genes. The detailed description of these approaches is beyond the scope of this chapter. Following is a list of transcription factors database and tools (Table 3).

**Plant Promoter Database (PlantProm DB): http://mendel.cs.rhul.ac.uk or http://www.softberry.com/**

PlantProm is a plant promoter database. The database represents a collection of annotated, nonredundant proximal promoter sequences for RNA polymerase II with experimentally determined transcription start site from various plant species [57].

**The Arabidopsis information resource (TAIR) motifanalysis software: http://www.arabidopsis.org/tools/bulk/motiffinder/index.jsp**

The motif analysis tool of the TAIR compares the frequency of 6-mer motif in promoter regions of query set of genes with the frequency of the 6-mer motif in the whole *A. thaliana* genome. A binomial distribution *p*-value is computed for each motif identified. The user can specify the size of the genes 5’upstream region to 500 bp or 1 kb. The tool does not account for multiple testing.

**TRANSFAC: http://www.biobase-international.com/pages/index.php?id=transfacdatabases**

TRANSFAC is an international unique database on eukaryotic transcriptional regulation [58]. The database contains data on transcription factors, their target genes and their experimental-proven binding sites in genes. Tools within TRANSFAC allow the users to automatically visualize gene-regulatory networks based on interlinked factor and gene entries in the database.

**AthaMap: http://www.athamap.de/index.php**

AthaMap is a database that organizes a genome-wide map of potential transcription factor binding sites in *Arabidopsis thaliana* [59]. AthaMap allows the user to test for the overrepresentation of transcription factors in a set of query genes. A colocalization tool performs combinatorial analysis to identify synchronized binding of pairs of transcription factors.

**PlantCARE (Plant Cis-Acting Regulatory Elements): http://bioinformatics.psb.ugent.be/webtools/plantcare/html/**

PlantCARE is a database of plant cis-acting regulatory elements and a portal to tools for in silico analysis of promoter sequences [60]. The database can be queried on names of TF binding sites, function, species, cell type, genes, and reference literatures. The program returns a list of entries with links to other information within the database or beyond through accession to TRANSFAC, EMBL, GenBANK, or MEDLINE.

**PLACE (Plant Cis-acting regulatory DNA Elements): http://www.dna.affrc.go.jp/PLACE/**

PLACE is a database of motifs found in plant cis-acting regulatory DNA elements, all from previously published reports [61]. In addition to the motifs originally reported their variations in other genes or in other plant species reported later are also compiled. The PLACE database also contains a brief description of each motif and relevant literature with PubMed ID numbers.

**Athena: http://www.bioinformatics2.wsu.edu/cgi-bin/Athena/cgi/home.pl**

Athena is a database which contains over 30 000 predicted Arabidopsis promoters sequences and consensus sequences for 105 previously characterized TF binding sites [62]. Athena enables the user to visualize and rapidly inspect key regulatory elements in multiple promoters. The software includes tools for testing the overrepresentation of TF sites among subset of promoters. A data-mining tool allows the selection of promoter sequences containing specific combination of TF binding sites. Athena does not adjust for multiple testing.

**AGRIS (Arabidopsis Gene Regulatory Information Server): http://arabidopsis.med.ohio-state.edu/**

AGRIS is an information resource for retrieving Arabidopsis promoter sequences, transcription factors and their target genes [63]. AGRIS integrates transcriptional regulatory information from multiple sources. Users can query the database with a gene name, gene symbol to retrieve its promoter along with other genes regulated by the same transcription factor.

## 7. ‘OMICS DATA INTEGRATION TOOLS

Various innovative and advanced technologies have allowed scientists to rapidly generate genome-scale or “omics” datasets at virtually every cellular level. These individual omics provide a wealth of information about living cells and organisms. However, it is only by integrating genomics, transcriptomics proteomics, metabolomics, and other recent omics types of data such as “interactomics,” “localizomics,” “lipidomics,” and “phenomics” that biologists can gain access to a more complete picture of living organisms and unexplored areas of biology. This challenging task requires a systems level approach to perform systematic data mining, cross-knowledge validation, and cross-species interpolation. Some investigators attempted the integration of genomic data and transcriptomic data [64], and the integration of protein-protein interation data and transcriptomic data [65] to analyze the dynamics of biological networks in yeast. The approach commonly used comprises three steps: (1) identification of the network that describes all interactions between cellular components from integrating various genome scale data; (2) decomposing the network into its constituent parts or network modules; (3) building a mathematical model that simulates biological systems for the purpose of simulation or prediction [66]. We describe below proteomics and metabolomics, and the potential of their integration with transcritomic data.

### 7.1. Proteomics

Gene mRNA expression profiling on a global scale in response to specific conditions is not sufficient to render the complexities and dynamics of systems biology. The ultimate products of genes are proteins. Furthermore, mRNA levels are not always well correlated with the levels of the corresponding protein [67] and one gene can produce several protein species. Indeed, proteins undergo a series of posttranslational molecular modifications such as glycosylation, phosphorylation, cleavage or complex formation may also occur that overall influence their function. Proteomics is the systematic large-scale study of proteins of an organism or a specific type of tissue, particularly their structure, function, and spatiotemporal distribution. Thus proteomics is an essential component of any functional genomics study aiming at understanding biological processes. The integration of transcriptome and proteome data has not always resulted in consistent results [68]. The methods and techniques used to measure the transcript level and the protein level may affect the results concordance. Nonetheless, the interpretation of the data in terms of biological pathways or functional groups gives better correlation of transcriptome with proteome in yeast [69].

Many plant proteomics databases have been constructed in recent years. As the plant model organism of choice, Arabidopsis proteome database contains more data compared to other species. Protein amino acid sequence databases and repositories for two-dimensional polyacrylamide gel electrophoresis as reference maps of proteomes are becoming popular as tools for analyzing and comparing the plant proteome. SWISS-2DPAGE is a two-dimensional polyacrylamide gel electrophoresis database (http://expasy.org/ch2d). PhytoProt (http://urgi.versailles.inra.fr/phytoprot) is a database of clusters of all the plants full-length protein sequences retrieved from SwissProt/TrEMBL. Proteins are grouped into clusters based on their peptide sequence similarity in order to track erroneous annotations made at the genome level. The database can be searched for any protein or group of proteins using protein ID or words appearing in protein description. Additional plant proteomics databases are provided in Table 4.

### 7.2. Metabolomics

Metabolomics is the study of all low molecular weight chemicals in a plant as the end products of the cellular processes. The metabolome represents the collection of all metabolites in an organism. Metabolic profiling provides an instantaneous snapshot of the chemistry of a sample and defines the biochemical phenotype of a cell or a tissue [70]. Similar to transcript level and protein level, the level of metabolites in an organism or a tissue is influenced by the biological context [71]. Thus measure of mRNA gene expression and protein content of a sample do not tell the whole story of biological phenomena unfolding in that sample. Although plant metabolomics is still in its infancy, recent advances in mass spectrometry have enabled the accumulation of metabolites data on a large scale for some species. Applications of metabolomics data to functional genomics are numerous. Metabolomics provide scientist with the ability (1) to characterize genotypes, ecotypes, or phenotypes with metabolites levels; (2) to identify sites within a genetic network where metabolites levels are regulated; (3) to analyze genes functions at the light of metabolites levels [70]. Currently, one of the most pressing needs in the fields of metabolomics for bioinformatics application is the creation of specific databases and biochemical ontologies. Such tools would help clearly describe the function, localization, and interaction of metabolites. However, databases imbedded in KEGG and AraCyc can be useful at least in part for the purpose of metabolites referencing.

## 8. CONCLUSION

The deluge of large-scale biological data in the recent years has made the development of computational tools critical to biological investigation. Microarray studies enables scientist to simultaneously interrogate thousands of genes throughout the genome. A great variety of tools have been developed for the specific task of drawing biological meaning from microarray data. Most of the tools available exploit prior biological knowledge accumulated in numerous publicly available databases in an attempt to provide a comprehensive view of biological phenomena. Table 5 summarizes the main features of each class of bioinformatics tool described. These tools differ in many respects and the guidance provided in this review will help biologists with little knowledge in statistics understand some of the key concepts. The integration of transcriptomics data with all other omics data is a challenging task that can be addressed by a systems-level approach.

## Figures and Tables

**Table 1:** Recapitulative list of GO annotations tools.

Tool name	Statistical model	GO abstraction level	GO visualization	Multiple testing	Type of array	Other annotation	OS
Onto-Express	hypergeometric, Fisher's exact test, binomial, *𝒳* ^2^	Available	DAG	Bonferroni, Holm, Sidak, FDR	172 commercial arrays	Chromosomal position	Any
FatiGO+	Fisher's exact test	Available	One level at a time	FDR	User-provided	KEGG pathways, SwissPROT keywords	Any
FuncAssociate	Fisher's exact test	Not available	Not available	Monte Carlo simulation	User-provided	Not available	Web-based
GoToolBox	hypergeometric test, Fisher's exact test or binomial	Available	One level at a time	Bonferroni	User-provided	Not available	Any
CLENCH2	Hypergeometric, binomial, *𝒳* ^2^	Static global	DAG	None	User-provided	Not available	Windows
BiNGO	Hypergeometric, binomial	Available, GOSlim	DAG	FDR, Bonferroni	commercial arrays	Not available
GoSurfer	*𝒳* ^2^	Lowest level	DAG	FDR	Affymetrix only	Not available	Windows

**Table 2:** Additional links for pathways databases on the internet.

Database name	Description	URL
PathDB	Biochemical pathways, compounds and metabolism	http://www.ncgr.org/pathdb
UM-BBD	University of Minnesota biocatalysis and biodegradation database	http://umbbd.ahc.umn.edu/
BIND	Biomolecular interaction network database	http://www.bind.ca/
BRITE	Biomolecular relations in information transmission and expression, part of KEGG	http://www.genome.ad.jp/brite/
PAJEK	Program for large network analysis	http://vlado.fmf.uni-lj.si/pub/networks/pajek/
DDIB	Database of domain interactions and binding	http://www.ddib.org/
DIP	Database of interacting proteins: experimentally determined protein-protein interactions	http://dip.doe-mbi.ucla.edu/
IntAct project	Protein-protein interaction data	http://www.ebi.ac.uk/intact/
InterDom	Putative protein domain interactions	http://interdom.i2r.a-star.edu.sg/
PSIbase	Interaction of proteins with known 3D structures	
Reactome	A knowledgebase of biological pathways	http://www.reactome.org/
STRING	Predicted functional associations between proteins	http://string.embl.de/
TRANSPATH	Gene regulatory networks and microarray analysis	http://www.biobase-international.com/pages/index.php?id=transpathdatabases

**Table 3:** Databases for transcription factors available on the internet.

Databse name	Description	URL
ACTIVITY	Functional DNA/RNA site activity	http://wwwmgs.bionet.nsc.ru/mgs/systems/activity/
DoOP	Database of orthologous promoters: chordates and plants	http://doop.abc.hu/
EPD	Eukaryotic promoter database	http://www.epd.isb-sib.ch/
JASPAR	PSSMs for transcription factor DNA-binding sites	http://jaspar.cgb.ki.se/
MAPPER	Putative transcription factor binding sites in various genomes	http://bio.chip.org/mapper
TESS	Transcription element search system	http://www.cbil.upenn.edu/tess/
TRANSCompel	Composite regulatory elements affecting gene transcription in eukaryotes	http://www.gene-regulation.com/pub/databases.html#transcompel
TRED	Transcriptional regulatory element database	http://rulai.cshl.edu/tred/
TRRD	Transcription regulatory regions of eukaryotic genes	http://www.bionet.nsc.ru/trrd/
AthaMap	Genome-wide map of putative transcription factor binding sites in *Arabidopsis thaliana*	http://www.athamap.de/
DATF	Database of *Arabidopsis* transcription factors	http://datf.cbi.pku.edu.cn/

**Table 4:** Proteomics databases available on the internet.

Databse name	Description	URL
RPD	Rice proteome database	http://gene64.dna.affrc.go.jp/RPD/
ANPD	Arabidopsis nucleolar protein database	http://bioinf.scri.sari.ac.uk/cgi-bin/atnopdb/home/
AMPD	Arabidopsis mitochondrial protein database	http://www.plantenergy.uwa.edu.au/applications/ampdb/index.html/
PA-GOSUB	Protein sequences from model organisma, GO assignement and subcellular localization	http://www.cs.ualberta.ca/~bioinfo/PA/GOSUB/
Swiss-Prot	A curated protein sequence database which strives to provide a high level of annotation	http://expasy.org/sprot/
AAindex	Database of various physicochemical and biochemical properties of amino acids and pairs of amino acids	http://www.genome.ad.jp/aaindex/
Prosite	Database of protein domains, families and functional sites, as well as associated patterns and profiles	http://www.expasy.ch/prosite/
PLANT-PIs	Database of information on the distribution and functional properties of protease inhibitors in higher plants	http://www.ba.itb.cnr.it/PLANT-PIs/
GeneFarm	Annotation of Arabidopsis genes and proteins	http://urgi.versailles.inra.fr/Genefarm/

**Table 5:** Main features of the types of bioinformatics tools used for the analysis of DNA microarray data.

Tools and resources	Goal	Methods
Class level functional Annotation	Determine a biological meaning to groups of related genes identified by microarray analysis	Overrepresentation test of gene ontology (GO) terms
Gene coexpression	Identify common expression patterns between genes in order to infer biological function	Correlation tests of gene expression
Gene network Analysis	Capture the interconnectedness of cellular components in order to explain biological phenomena	Systems biology approach
Gene network reconstruction	Develop models for gene regulatory networks	Bayesian inference theory Mutual information theory
Network visualization	Display a simplified view of large amount biological components and their interactions	Graph theory
Network exploration	Associate network nodes and edges with biological information	Incorporate heterogeneous data from various databases
Biological pathway resources	Map biological pathways information into inferred network	Collect and process information from pathway databases
Transcriptional regulation analysis	Identify transcription factors that regulate gene expression	Overrepresentation test of regulatory motifs in promoter regions of related genes
